# The Prevention of Syphilis*Read before the Buffalo Medical and Surgical Association, Tuesday evening, Oct. 2, 1883.

**Published:** 1883-12

**Authors:** Eli H. Long


### The Prevention of Syphilis.*

By Eli H. Long, M. D.

In considering the subject of the prevention of syphilis, we have before us a matter which is of interest, not only to the members of the medical profession and capable of being discussed by them, but one which is considered and discussed by educated minds in all of the various stations and pursuits of life. Aside from its being a matter of study to the physician, as is the lessening of any disease, it presents questions to the sanitarian, to the philanthropist, and to the moralist, more than does perhaps any other one subject that engages our attention. I have selected this subject for discussion this evening, not that I hope to add anything new to the much that has been said from the various standpoints, but because I consider the matter one of great importance to all, and believe the discussion of it to be a means of education toward devising methods for combating the evil, which time will doubtless show to be a public necessity.

In the preparation of this paper I am indebted to Dr. J. Wm. White of Philadelphia, from whose excellent and thorough dis-

* Read before the Buffalo Medical and Surgical Association, Tuesday evening, Oct. 2, 1883.

cussion of the matter, in an address to the Philadelphia County Medical Society, I have gleaned numerous interesting facts and also the bulk of the statistics that I present.

It is unnecessary to speak of the characteristics of the disease, for they are known by you all; therefore, they will be referred to only where necessary in order to complete or confirm thoughts expressed.

It is a fact which we note and are thankful for, that our civil government provides for our protection against invasions by cholera and like scourges from abroad, and seeks to limit the spread of epidemics at home; but we must deplore the fact that little or nothing is done to stay the progress of this prevalent malady, which, if does any, merits the name of "the scourge of humankind." We are glad that the thousands of dollars are being expended to remove our Hamburg canal nuisance, but we must be content to think with horror of its neighbor, the infected district, whose poison numbers annually many more victims than do the emanations from the former, to say nothing of it as a hot-bed of vice and immorality.

It is easy enough to make such comparisons, and to speculate that syphilis, the world over, carries more people to the grave than does cholera or many another dread disease, but to suggest a practicable remedy is a matter of greater difficulty. It is one of the problems of civilization, which time, experience and education must help us to solve. It is unfortunate that a disease which is the source of so much misery should be placed under seal, as it were, by the existing state of society, so that it is forbidden that it should be spoken about in respectable hearing, or discussed by the press, or alluded to by the platform and pulpit, as almost any other evil of like prevalence may be.

It is believed by those who have taken pains to gather statistics that at least two millions of our population of fifty millions are affected with syphilis in some one or more of its various phases. This would be an average of one in each twenty-five persons. The truth of these figures may, at first, be doubted by those of us who may be unacquainted with hospital and dispensary practice, who have not the opportunity of observing the frequency of the disease as it occurs throughout the land; but any one studying the cases presented at the dispensaries and almshouses of our larger cities, cannot fail to notice the great number who are suffering from this disease.

History gives us no positive date of the first appearance of syphilis. It is supposed by some to have existed in the time of Moses and Job, 1520 B. C., and that later it was described by Hippocrates, 460 B. C., Galen, A. D. 130, and others. We have reliable history to the effect that it was very prevalent in Europe in the latter part of the fifteenth century, and in the sixteenth century it was recognized as being caused by a specific virus. Since then it has spread throughout the world, gaining entrance into the best society, afflicting alike the rich and poor, and becoming the life-long, dread companion of multitudes, to many of whom grim Death comes a welcome guest.

As to the increase of the disease, I am led to believe that a disease so easily communicated, so secret that the unsuspecting and innocent cannot know of its existence until brought into sad relationship with it, and withal, a disease unrestricted in its spread by sanitary or legislative measures, cannot fail to increase in a progressive ratio. Touching this question, I will quote from the writings of the late Mr. Acton in 1869, who says: "I doubt whether venereal complaints, although evidently more severe formerly, were ever more common than at present, or whether, since syphilis was first treated in hospitals, the large proportions here noticed, namely, two out of three out-patients at the Free Hospital, nearly one in two at St. Bartholomew's, one out of every three at the Dreadnought, one out of four in the army, one out of seven in the navy, at any former period suffered from venereal disease; and yet many believe the disease is declining. That such is not the case, if number be any criterion, must be admitted by all who weigh well the above statistics, and compare them with the statements met with in nearly all the books that have treated of syphilis."

It is plainly a difficult matter to obtain reliable statistics of this disease, of which many victims do not come under the observation of physicians, being treated by quacks and drug clerks. Therefore, from partial statistics it is necessary to estimate, in order to obtain an approximately correct result.

In the British navy, during 1879, in ports protected by laws one sailor in every thirty or thirty-five contracted syphilis. In ports not protected by law, one in every ten or twelve acquired the disease. In the United States navy one sailor in every twenty is infected each year; how many of the nineteen others already have it we cannot say. Dr. Sturgis, after a careful study, concludes that there are in New York City, at any one time, from sixty to seventy thousand venereal cases,—about one in every fifteen of its population. After comparing figures taken from dispensary and hospital reports and from the statistics for the mercantile marine, he submits calculations which would give the number of venereal cases as one in each fifteen inhabitants, and the cases of syphilis as one in each eighteen or nineteen. It is estimated that in Great Britain 1,652,000 persons are annually infected with syphilis. In Prussia the government has been active to lessen the spread of the disease, and in the Prussian army the number of venereal cases are reported to be only fifty-four per one thousand. In Northern Africa syphilis is very common, and it is reported that the majority of the negroes of Western Africa are sooner or later infected. At Cape Town, in 1868, in six months 1,865 persons furnished 315 cases of syphilis. These are a few of the many reports which go to show the prevalence of the disease throughout the world.

After considering the presence and extent of the disease, we naturally turn our attention to the causes; but as the very name of syphilis or venereal disease suggests to us the predominant, direct cause, we will not dwell upon *it*, but pass to the consideration of a more remote, but very prevalent causative influence which is—ignorance. Although in the great majority of cases the contraction of syphilis is the result of vicious conduct, there are still many instances, a sad chapter in the history of mankind, where the pure in life innocently receive the disease, and suffer therefrom the accompanying miseries, and oftentimes drag out a weary existence, suffering for the sins of others.

What can be sadder than to see a respectable married woman suffering with symptoms for which she is unable in anywise to account, but which, at a glance, are known to the physician whom she consults, to be secondary or tertiary symptoms of syphilis, which was innocently acquired from an immoral husband, whom she supposes to be true and virtuous? Who of us has not known of a woman shrinking from society because of deformity unaccountable to her, such as the loss of the palate or other bones of the face, the result of similar infection? Think of the woman suffering with syphilitic ulcers, or with severe nocturnal headaches, the cause of which you dare not reveal to her. Think of the number of innocent children born with hereditary syphilis, who are doomed to an early death or a miserable existence. Think of the numerous instances in which the disease is communicated by the kiss of affection, by the handling of infectious toys or clothing, by the instruments of the dentist, and in the case of children by means of syphilitic wet-nurse or servants.

We cannot doubt that to no small extent the spread of the disease is due to want of care, the result of ignorance, on the part of those already infected. If a man having the initial lesion understands the gravity of the disease he has contracted, the activity of the poison thereof, and the liability of infecting others and bestowing upon them the train of dire consequences, can we not believe that he will restrain his desires during the infectious period of the disease, rather than endanger the health and happiness of those for whom he possesses affection or regard? Will he not be more careful about his personal cleanliness, and in all his social intercourse avoid any possibility of communicating the disease to others? Doubtless there are persons so selfishly degraded and inhuman as to furnish exceptions to the above, but will it not apply to perhaps the majority?

It is an established rule in the history of venereal diseases that the primary inconvenience of the disease bears an inverse relation to its real gravity. Thus, gonorrhœa, ordinarily the most harmless of venereal diseases, causes the patient at first the most pain and inconvenience. Secondly, the venereal sore or chancroid causes less primary trouble, but gives rise to suppurating buboes, offers a chance for phagedenic sores, and, according to some good authorities, a possibility of constitutional infection. Lastly, the chancre, which appears to the ignorant patient of little significance, and which, in the case of filthy persons, especially women, may remain undiscovered, exemplifies the rule fully as you all know, being the herald of a disease most loathsome and terrible.

From this rule we can glean an explanation answering for much of the rapid spread of syphilis. Take for example a person ignorant in these matters who has the initial sore, small, hard, painless, and seemingly of little moment. From his view of the case he sees no necessity for seeking medical advice any more than he would for the presence of a sliver in his finger. As the sore causes him little inconvenience, he is not obliged to deny himself lustful indulgence, and he is apt thereby to infect others before the appearance of graver symptoms. Let us suppose that one of the persons infected by him be an ignorant, careless public woman. She may not be aware of the sore when it appears upon her, and especially not of its character. As her shameful business is a source of sustenance to her, she will doubtless continue therein through the primary and early part of the secondary stages, suffering for the sake of money the slight inconvenience she may feel, until induced or compelled by graver general symptoms to suspend her occupation and seek advice.

We cannot tell how many persons she may have infected during all of this time, but the number would probably reach its fives, or tens, or more. But let us suppose the number to be five, and let them carry the virus their various ways, and communicate the disease to others, as would naturally occur, and I leave it to each one of you to multiply for yourselves the amount of disease and misery which might be inflicted upon the community and the world from this one source of infection. Is it not plain that, with wise restrictive measures and proper knowledge, instead of the unbridled liberty of ignorance, much of this evil might have been averted?

Perhaps the saddest part of this history would be the sufferings of the innocent and the transmission of the disease to the unborn.

This is indeed a woeful but true picture to gaze upon. And shall we allow the evil to grow, fostered by an ignorance which we can in a measure dispel? And shall we allow the lawless liberty that prevails to remain, a source of danger to the innocent and uninstructed, when we know that its restriction in other lands, by other nations, has been a boon to the people? It remains for us as guardians of the public health and sanitary instructors, to agitate the matter and to devise means to quench this ever-increasing and devouring evil.

In the consideration of means of prevention, I will first call your attention to the subject of education.

The true, unprejudiced philanthropist must deplore the present state of society and the family which clothes them superficially with the garment of false modesty, and prevents the dissemination of proper knowledge on many important topics. To a greater or lesser degree is *this* accountable for the gross ignorance upon the subject at hand.

It is plainly proper to avoid conversation relating entirely to sexual matters, between mere friends and acquaintances, upon the ground of modesty, and also because uncalled for; but concerning the dreadful disease under consideration, which from its *nature* possesses an interest to the entire community as a whole, and to the people individually, should rational conversation be considered an impropriety merely from the fact that its most frequent mode of transmission is through vicious and shameful conduct?

In the social world this existing state effectually bars the discussion of the matter by the press, the platform and the pulpit. Were it not far better for humanity, if our newspapers and journals contained sound articles instructing the public upon topics of vital importance, although savoring of a private nature, rather than teem with extended accounts of scandals, trials for seductions, murders, rapes, etc., written up in language especially designed to suit the popular mind? The latter class of reading cannot fail to beget in the minds of the sensual, the immature, and the curious, a most unwholesome literary taste, which destroys the relish for literature that tends to elevate and refine. The former class of reading, although calling forth some objections, would instruct the people in regard to facts of great importance to themselves. It would furnish knowledge which would be a safeguard to them against previously unknown dangers; knowledge that would enable them to conform more fully to the rules of health; and knowledge that doubtless would be a means of raising the standard of morality, and creating in the minds of the people an appreciation of the value of the advice and labors of scientific men for the good of the race.

Could not the platform be utilized to educate and mould public opinion in regard to this evil? Why not encourage the delivery of lectures by able men and women, to audiences exclusively of either sex? Much could be done in this way to counteract the false teaching of the pamphlets and lectures of quacks, which, with mercenary aims, are placed within easy reach of all, and in place would be furnished sound information, given with an honorable purpose.

Now, as to our duty personally as physicians, many are the opportunities afforded us to instruct. Those who look to us for care in sickness, gladly receive instruction as to means for maintaining health; and as guardians of health, are we not grossly neglecting our duty if we allow the spread of disease, when by timely instruction we may prevent it and all of the misery and pain that it brings? We can recommend the perusal of scientific books upon special subjects, written for the people. Such books are gladly read, and with profit. We can instruct parents, that they in turn may instruct their children as to personal care, and as to the danger of intimacy with persons of questionable character. By such means children would receive the instruction from their rightful teachers.

In the family we find the most natural school existent. It is there we learn, not only reading and writing, but there are moulded the essential elements of our life. There is our character built up, and there we receive our first lessons in the joys and sorrows, blessings and duties, and virtues and follies of life, which fit us for our relations with the world in maturity. This all results largely from the precepts and examples of our most natural teachers, our parents, the foundation of whose interest for our welfare is laid in love. Who of us have listened to the counsels of a kind father without feeling that the motive thereof was a love that desired only good for us?

If, then, the home is the place of essential preparation for life and duty, we ought to look to it for aid in the spread of proper knowledge of a special character. Shall it not be proper, and even a duty, for fathers and mothers to instruct their sons and daughters in useful knowledge of a private nature? It would be far better for a son to learn from a confiding father the dangers, debasement and sinfulness of youthful abuses and indulgences, than from personal experience or association with impure companions. If only the dangers to health from indulgence were kindly represented, would it not be a guard to the virtue of many a young man? But much more would the confidence shown by the parent, and the appeal to true manliness, be incentives to purity of life with its freedom from the disease we are considering.

What applies to young men will equally apply to young women. Small chance do our sisters have of knowing the extent to which vice and immorality exist in society, and as well the low character of many whom society compels them to associate with. Instead of a knowledge which would be to them safety, they must remain in an ignorance more or less dangerous, unless it is dispelled by the counsels of a sensible father or brother.

If parents and guardians would thus regard the health of the young in this particular, as well as their school and business interests, and add to their duties that of giving the needed instruction to that end, I think we could reasonably expect a decrease in venereal disease, with a decided rise in the standard of morality of youth.

Doubtless we will all agree that the object set forth in this section is most desirable, and perhaps many of you would endorse the means recommended for its attainment; at the same time we know that the difficulties in the way of their satisfactory operation are many. We have a public opinion, fostered largely by ignorance, to fight against. We find many parents not sufficiently intelligent or interested to do their part. We find among many a sad want of self-respect and cleanliness, which are conducive to health and morality.

We could not hope to attain to any appreciable result in a short time; but, evidently, if it is desirable to instruct the people, and mould public opinion, in order to the more effectual suppression of vice and immorality which beget disease, the place for such a move to begin is with the medical profession, the members of which are the most capable teachers. Let us interest ourselves in the matter, and by discussions in our meetings and in our journals let us seek to interest the profession at large, and going further, to enlist in our cause the liberal-minded men and women who appreciate the necessity of the work. Let us plant the seed by giving wholesome instruction to the heads of families of which we have the care, leaving it to grow and increase, believing that future generations may thereby be made better and happier.

We now pass to the consideration of another, a more direct and positive means of prevention of syphilis, viz., the legislative prevention.

The subject of legislative interference is one that has been under discussion for many years, and there have been many able men who have favored legislation, and many who have condemned it.

There have been preventive laws enacted in different countries, and also in parts of the United States, and perhaps many more proposed, and in most cases results most pleasing to the sanitarian have followed their adoption and enforcement.

In England the "Contagious Diseases Acts" were passed in 1865, providing for protection against venereal disease in the military and seaport towns, and were intended for the benefit of the soldiers and sailors. They provided that, upon sworn information of a superintendent of police that a prostitute was suffering from venereal disease, she was subjected to examination, and if found necessary, she was detained in a hospital for from three to nine months. These acts were since amended and made more effectual.

In France, legislation to this end is not limited to naval and military points, but extends to the whole civil population. In our own land there has been, in the past, a law in successful operation in St. Louis, which, after a time, was rendered extinct through the efforts of a class of moralists, who considered the law as nothing more than a means of licensing and encouraging prostitution. The law, when in force, gave authority to the board of health and police, for the registration of all prostitutes, for their periodical examination by physicians appointed for the purpose, and when found with venereal disease, for their isolation in the female hospital. The women were compelled to pay weekly a certain sum, which was used entirely for their benefit in helping to support their hospital.

In the discussion of the great questions pertaining to health and morality, like the one before us, we meet with such diversity of opinion among the friends of humanity, that we are often perplexed to define the line of right thought and action.

There is a class of philanthropists whose minds are governed entirely by ideas of morality; who have their ideal of a moral community; an ideal so grand that they will never see its reality. Such men will give such scope to their strict ideas of morality, that they will ignore the necessities of the community as it exists. They will seek to elevate at a bound a lot of degraded human beings, to a moral status nigh to that of divinity; or, failing to do this, will leave them, perchance, in their depravity, without extending the hand of sympathy for fear of defilement. Such men will strictly condemn any plan for the suppression of vice and disease which will allow the least liberty demanded by the existing grade of civilization, or afford the least protection to the unfortunate beings who propagate vice, and who have been led to sin, oftentimes, through circumstances which entitle them to a measure of sympathy from their more fortunate fellow-beings.

There is another class of persons who take a broader view, and look to the demands of the people as we find them; who appreciate that many men are possessed of perverted consciences and a low degree of stability; that the animal passions are stronger in some persons than in others; that the majority of men live for the present enjoyment of life only, and that it is the lesser number who place themselves under restraint in the present life, and educate themselves to enjoy the future life. They see that the highest possible plane of morality to which they could at present elevate the masses, must fall far short of the high standard maintained by the most conscientious. They see the necessity of protecting the people from the ravages of this most terrible disease, although requiring for its accomplishment means that apparently savor slightly of sensual liberty.

With this latter class have originated the various laws for the prevention of syphilis, and, imperfect and objectionable as these laws may seem, are we not forced to believe with them, that sooner or later such legislation will become a public necessity? Knowing men as we do, can we deny that prostitution is an evil, necessitated by the present immorality of the masses, and as such entitled to the protection that such laws would afford? Prostitution is plainly a guardian of virtue to the better classes of the people. Were it abolished, multiplied would be the cases of rape, seduction and secret prostitution. Many homes would be polluted that now are pure, and the abode of happiness. The passions of the degraded would find vent somewhere, and doubtless greater sorrow and danger to the unprotected would exist under such abolition than does at present. For example, Bayard Taylor writes concerning Stockholm: "It has been called the most licentious city in Europe, and I have no doubt, with perfect justice. Nearly one-half the registered births are illegitimate, to say nothing of the illegitimate children born in wedlock. I have never seen any place where licentiousness was so open and avowed, and yet where the slang of a sham morality was so prevalent. There are no houses of prostitution in Stockholm; the city would be scandalized at the idea of allowing such a thing. A few years ago two were established, and the fact was no sooner known than a virtuous mob arose and violently pulled them down." Another illustration: Since the Reformation, the city of Berlin has thrice attempted to abolish all such houses, in 1717, 1796 and 1845, and each time with harmful results. The following is from the official report of the last attempt:

"The licensed houses, twenty-six in number, were closed in opposition to the remonstrance of the police. Their three hundred inhabitants, as well as all other females without ostensible means of support, were banished. But unnatural offences, self-abuse, secret prostitution, and illegitimate births became so common, and syphilis so much more than ever severe and frequent, that Gen. Von Wrangel, who was in command of the troops stationed at that post, was induced to make a forcible appeal on behalf of the army's health, against the quasi improved order of things. The number of females who had entered the venereal hospital had at this time risen thirty-three per cent., and that of males about thirty-one per cent."

The high tide of emigration to our country must remind us that our population is increasing, and that this influx is bringing us masses of ignorant people who need laws for their control and protection rather than moral suasion.

The principal arguments urged against legislative prevention are the following:

"That prostitution would thereby be legalized and encouraged."

"That there would be an increased patronage of houses of prostitution from the sense of security from infection that the laws would foster."

"That the periodical examination of the women would be degrading and improper."

The only means of satisfactorily proving or disproving the soundness of these objections, is by noting the results of the working of the laws where in force; and to this end I will present a few of the many reports summarized by Dr. White in his report:

"In France, the proportion of venereal disease among the troops was only one-fourth of that found in the English army before the operation of the Contagious Diseases Acts in the latter country."

"In Paris, among the registered prostitutes, one in twenty-five has venereal disease; among those who are unregistered, but under surveillance, one in four. In Bordeaux, the proportion of registered women found diseased has never exceeded 18 per 1,000 during the last eight years, while of clandestine prostitutes 350 to 500 in 1,000 were affected. Almost the same figures apply in Rotterdam."

"In Belgium and Prussia, since the State has undertaken the duty of regulation, the following facts have been noted and officially reported:

1. Clandestine prostitution has diminished.

2. The diseases have assumed a milder character, and the average period of stay in hospital has been correspondingly reduced.

3. Unregistered or clandestine prostitutes are affected most frequently, and with the severer forms of syphilis.

4. The average number of men in military service who acquire disease is much less.

5. The women exhibit a better sanitary condition, greater cleanliness and self-respect, than ever before, and many of them are annually reclaimed.

6. The form of disease most diminished or modified is that in which society in general is most concerned, namely, the syphilitic."

Concerning Asia, the Surgeon-General of the Navy is authority for stating: "The amount of venereal disease on the Asiatic station has fallen from 425.8 per 1,000 to 112.1 per 1,000,—a difference of 313.7 per 1,000, due to the examination of prostitutes practised at Hong Kong and in Japan, and the seclusion of infected women in lock hospitals."

In St. Louis, at the expiration of two years, the chief of police reported that the number of public women had uniformly decreased each year (46 per cent. in the first eight months); that soliciting upon the streets had been almost entirely discontinued; that a considerable number of women had been reclaimed; that private prostitution had been materially checked; that juvenile prostitution was almost wholly removed; that deaths among the registered women had largely decreased.

In Nashville, during 1863, 1864 and 1865, laws were adopted for the benefit of the troops stationed there, and the prostitutes were examined once every ten days. Col. Fletcher reports the following results:

1. The amount of venereal disease was markedly lessened.

2. The women who were at first rebellious, became quite reconciled to the system.

3. It was self-supporting, the fees paying the expenses of the hospital.

The following extracts from report of Commissioners of Metropolitan Police for 1880, published in the London *Lancet*, May 7, 1881, are interesting:

"The removals from the register are of great interest, and are thus classified. 927 left the district, 67 married, 262 entered homes, 629 returned to their friends, and 22 died. The mortality for the past five years has remained steadily at less than 6 per 1,000. The amount of obstruction to the acts on the part of the women themselves was so small that in only eleven cases was it necessary to apply to the magistrate for an order." "With regard to the work of reclamation, the beneficent value of which can scarcely be estimated, we find 93 young girls under eighteen, 86 women under thirty, and 7 above that age have been rescued from bad company and immoral places. Of these, 13 were under fifteen years of age. Besides these, 174 had commenced an immoral life, but abandoned it on being cautioned, and are therefore not registered. Of these, 4 were under fifteen and 45 under eighteen years of age."

In the face of such facts and figures, I have no desire to comment upon the propriety of such laws, nor upon the results of their enforcement, but will leave you to draw your own conclusions and form your own opinions concerning the subject presented. But in conclusion, turning slightly from my subject, I wish to express an opinion, that if any work within the scope of the philanthropist is deserving of our praise, it is the work of benefiting and reclaiming fallen women, many of whom dire necessity has driven to a life of shame. Such women deserve our sympathy, and as reports show that such work has been carried on successfully under the legal system, all must agree that at least in this particular the laws have been beneficial.

The establishment of lock hospitals, where these unfortunate women can receive treatment, is a humane provision that many of our cities lack. If we admit that prostitution is an evil necessity of the age, then that evil will be best controlled by recognizing it as a necessity, and dealing with it intelligently upon sanitary and moral grounds.

As a closing paragraph, permit me to present a graphic picture from the pen of Mr. Lecky in his "History of European Morals." After a consideration of the causes of prostitution, he says: "Under these circumstances, there has arisen in society a figure which is certainly the most mournful, and in some respects the most awful, upon which the eye of the moralist can dwell. That unhappy being whose very name is a shame to speak,—who counterfeits with a cold heart the transports of affection, and submits herself as the passive instrument of lust,—who is scorned and insulted as the vilest of her sex, and doomed for the most part to disease and abject wretchedness and an early death,—appears in every age as the perpetual symbol of the degradation and the sinfulness of man. Herself the supreme type of vice, she is ultimately the most efficient guardian of virtue. But for her, the unchallenged purity of countless happy homes would be polluted, and not a few who, in the pride of their untempted chastity, think of her with an indignant shudder, would have known the agony of remorse and of despair. On that one degraded and ignoble form are concentrated the passions that might have filled the world with shame. She remains, while civilizations rise and fall, the eternal priestess of humanity, blasted for the sins of the people."

